# Concurrent management of nasal bone expansion from nasal polyposis (Woakes' disease)

**DOI:** 10.1002/lio2.866

**Published:** 2022-08-09

**Authors:** Alexander Dickie, Brian Rotenberg, Leigh Sowerby

**Affiliations:** ^1^ Department of Otolaryngology ‐ Head and Neck Surgery London Health Sciences Centre London Ontario Canada; ^2^ Department of Otolaryngology ‐ Head and Neck Surgery St. Joseph's Hospital London Ontario Canada; ^3^ Department of Otolaryngology‐Head and Neck Surgery Western University London Ontario Canada

**Keywords:** chronic rhinosinusitis with polyposis, closed reduction, endoscopic sinus surgery, nasal bone, rhinoplasty, Woakes' disease

## Abstract

**Background:**

Woakes' disease is the eponymous name for severe chronic rhinosinusitis with nasal polyposis (CRSwNP) leading to thinning and expansion of the nasal pyramid. The endoscopic treatment of the sinus disease, while extensive, is standard practice for the rhinologist. Management of their external nasal deformities, for many, is not. Simultaneous closed rhinoplasty in these patients is straightforward, easy to perform and achieves an excellent esthetic outcome.

**Methods:**

Three patients with CRSwNP and notable nasal pyramid expansion are reviewed. All patients had eosinophilic disease, with two having NSAID‐exacerbated respiratory disease (N‐ERD). All three patients underwent full house endoscopic sinus surgery from May 2018 to September 2019 along with simultaneous closed rhinoplasty. Two of these patients required only external digital pressure to fracture the nasal bones for gentle Boies elevator repositioning, while the third had osteotomies with minimal force to aid reduction.

**Results:**

Postoperatively, patients had excellent nasal airway symptom improvement, and the cosmetic results following rhinoplasty demonstrated normalization of symmetry, profile, and contour of the nose with high‐patient satisfaction.

**Conclusion:**

Based on our experience, simultaneous rhinoplasty on the thinned nasal bones of Woakes' Disease patients is not only easy to perform, but provides excellent cosmetic and functional results by allowing bone to remodel in the appropriate position, and avoids a second‐stage rhinoplasty.

## 1 BACKGROUND

Edward Woakes, a renowned otologist, rhinologist, and pioneer of the ethmoidectomy in the mid‐to‐late 1800s, collected many cases of what he then described as “necrosing ethmoiditis.” He published in 1887 on sinusitis with associated expansion of the nasal bones and superior maxillae. In the 1920s, the disease was further characterized by French and Italian otolaryngologists, and by the 1950s “Woakes' disease,” became defined by bilateral nasal polyposis with hyperplastic expansive deformities of the nasal bone, along with resorptive changes of the bony ethmoid and osseous fibrosis.^1^

In contemporary rhinology, chronic rhinosinusitis with nasal polyposis (CRSwNP) is a common presentation managed effectively with a combination of medical and surgical therapies. Because of the relative ease of access to appropriate care, complications such as Woakes' disease have become increasingly rare.

The aim of this study was to present the care of these patients from the otolaryngologist's perspective and demonstrate the ease and efficacy of closed nasal bone reduction using manual external compression in patients with nasal bone expansion and rarefication.

## 2 METHODS

A review of the presentation, management, and outcomes for three patients with Woakes' disease was performed. Consent was obtained for the publishing of photographs and images. The Research Ethics Board (REB) of Western University, London, Ontario, Canada classifies this study as a case review, and has confirmed that formal REB review is not required.

## 3 OPERATIVE TECHNIQUE

The patient is positioned supine with image guidance for revision surgery. The nasal septum, turbinates, and lateral wall are infiltrated with 1% lidocaine with 1:100,000 epinephrine and the nasal cavity is decongested with a 1.2% cocaine and epinephrine solution. Complete endoscopic sinus surgery (ESS) was then performed in the standard fashion, tailored to the patient presentation and severity of disease.^2^ Care is taken to remove all polypoid disease adjacent to the inner mucosal surface of the nasal bones back to the olfactory cleft and middle turbinate. Triamcinolone‐soaked Nasopore® absorbable packing is placed in the middle meatus bilaterally.

Once the endonasal portion of the surgery is complete, the nasal bones can be easily reduced with digital pressure to the bilateral external nose. They can be manipulated using a Boies elevator to gain desirable contour and symmetry of the nasal pyramid. If not easily fractured with digital pressure, lateral osteotomies are performed using a guarded osteotome to mobilize the expanded nasal bones. A nasal cast is then applied on top of mastisol and steri‐strips. Patients were discharged home from the postanesthetic care unit and seen in 1 week for cast removal and debridement of the middle meatal cavity as indicated.

## 4 PATIENTS

### 4.1 Case 1

**FIGURE 1:**
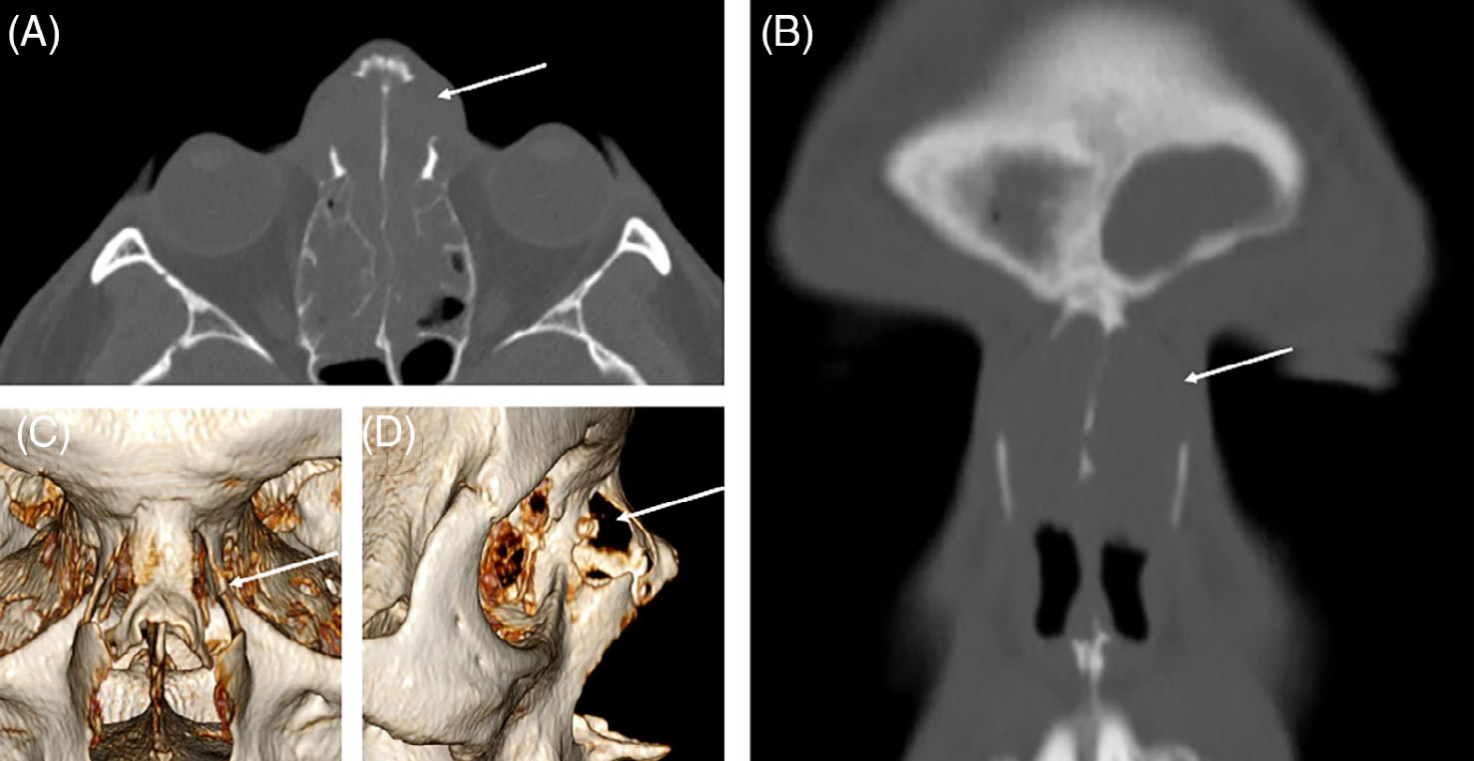
Case One: Preoperative axial (A) and coronal (B) CT planes demonstrating near‐complete rarefication of the lateral nasal bones (white arrow). Three‐dimensional reformats in frontal (C) and lateral (D) projections elucidate the bony deficit

**FIGURE 2:**
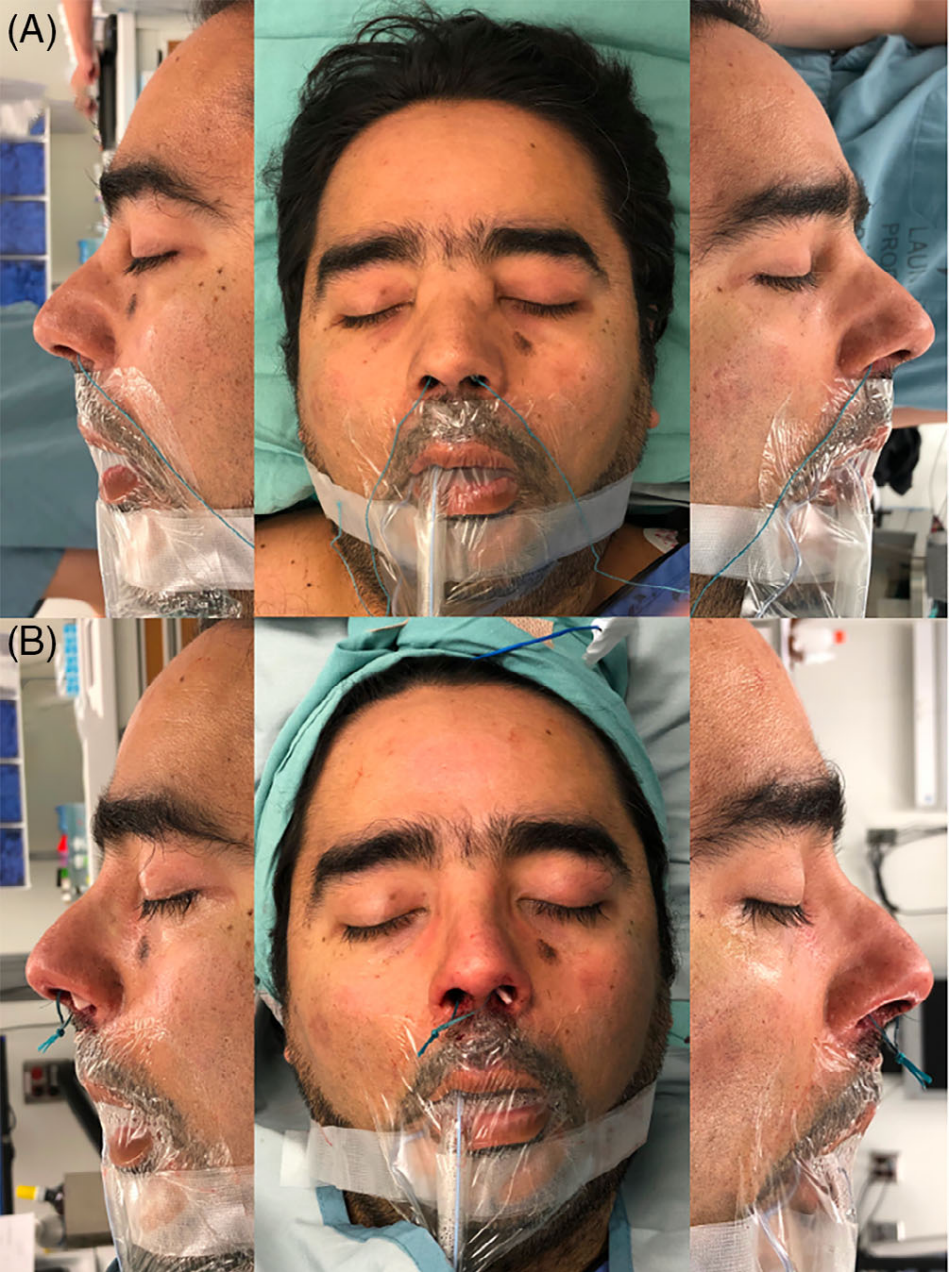
Case one: intraoperatively before (A) and after (B) closed rhinoplasty

**FIGURE 3:**
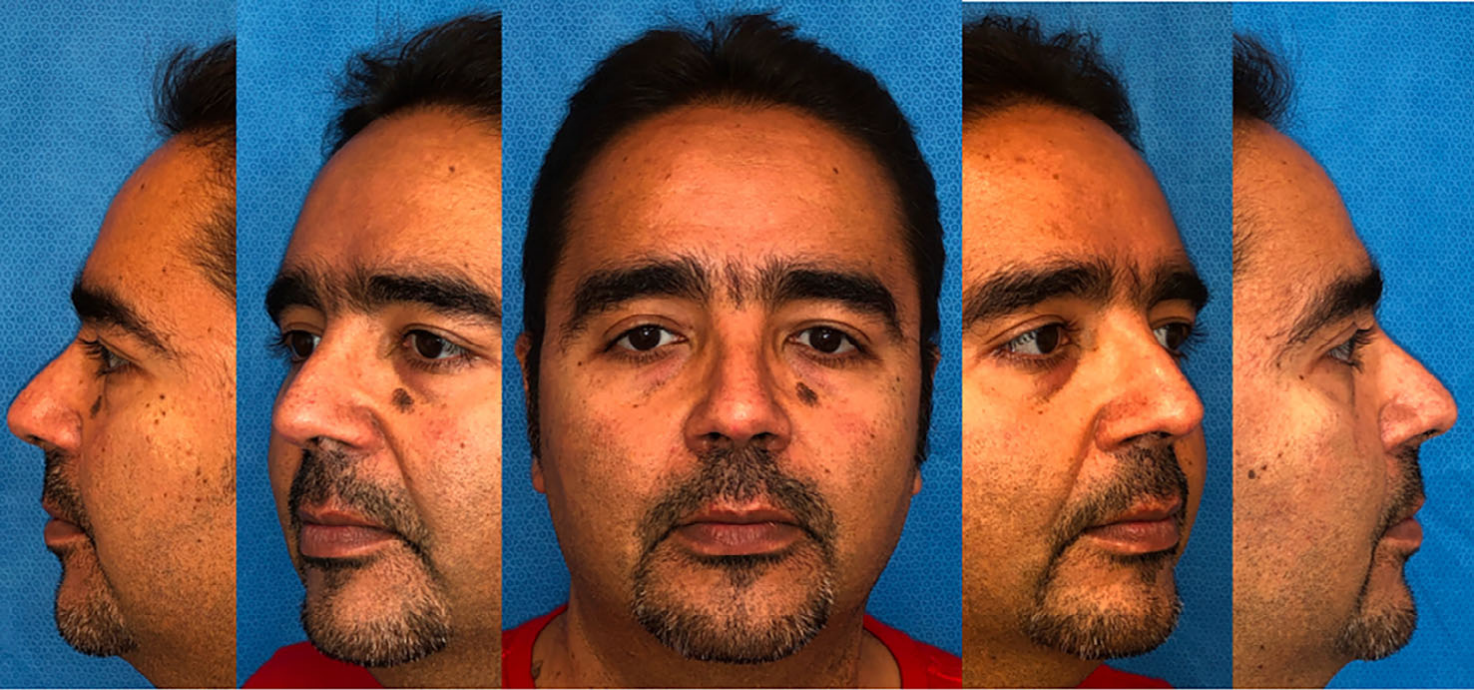
Case one: Postop follow‐up at 4 months

A 42‐year‐old male with N‐ERD from Egypt and a history of sinus surgery in 2008 presented with nasal congestion and 2 years of progressive swelling in the medial canthal area such that he could no longer wear eyeglasses. Previous attempts at ASA desensitization had resulted in significant shortness of breath. Physical examination demonstrated bilateral grade IV polyposis and symmetrical nasal pyramid expansion. A CT scan of his sinuses revealed partial maxillary and sphenoid, but complete ethmoid and frontal sinus opacification with thinning and lateral displacement of the nasal bone (Figure 1). Bloodwork confirmed elevated serum IgE and eosinophilia. He underwent complete revision ESS with image guidance and a closed rhinoplasty by external digital pressure to reposition the nasal bones. (Figures 2 and 3).

### 4.2 Case 2

**FIGURE 4:**
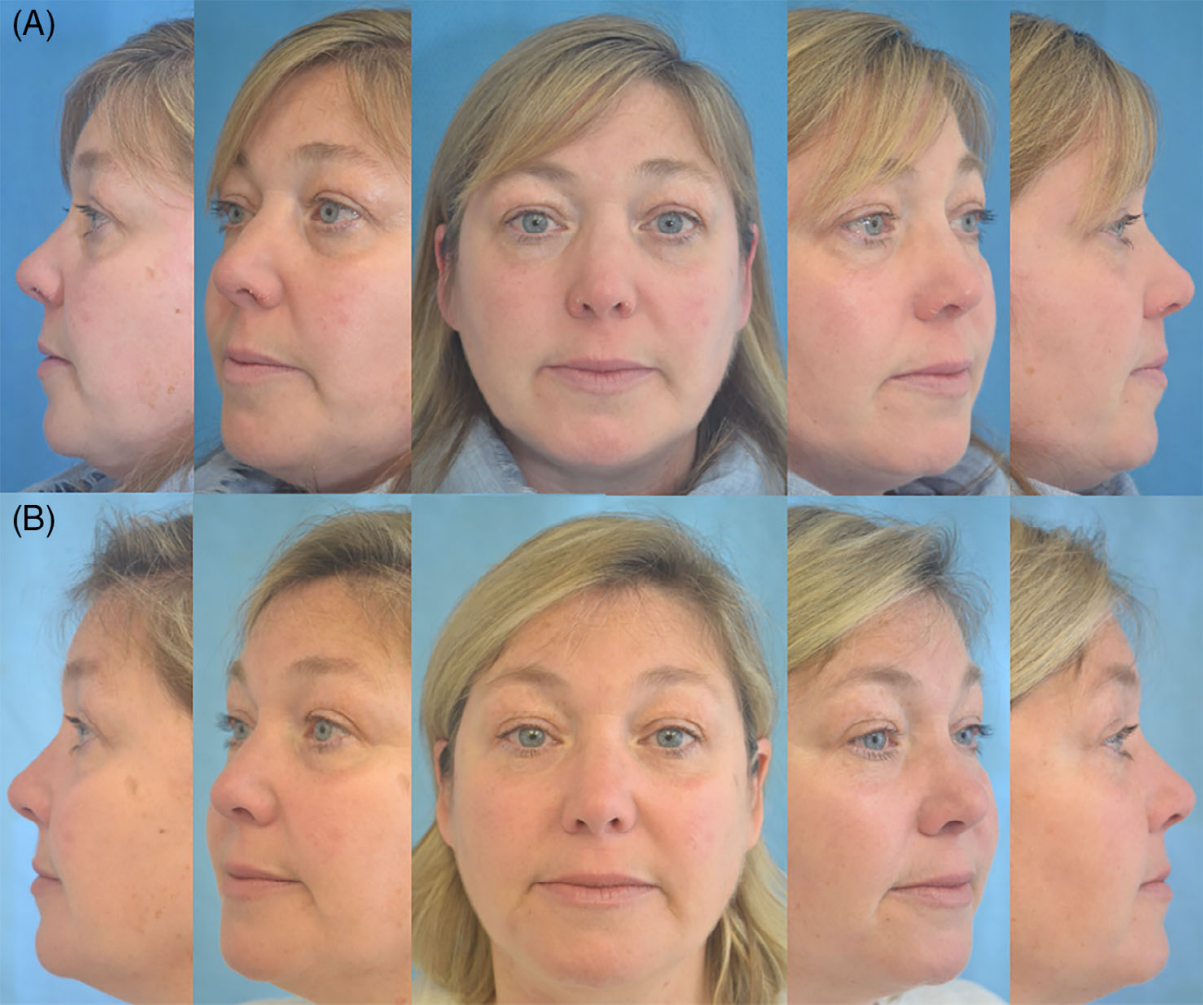
Case two: Preop (A) and 6 months postop (B)

A 49‐year‐old female with N‐ERD and a history of five previous sinus surgeries, most recently 3 years previous, presented to tertiary care with a 3‐month history of worsening congestion, anosmia, and facial pressure after switching intranasal corticosteroids. Exam revealed bilateral grade 4 polyposis and a CT scan confirmed complete frontal and ethmoid opacification. She expressed hesitation to proceed with surgery and initially had significant improvement on prednisone, budesonide nasal rinses, and a low‐salicylate diet. After 6 month, on routine follow‐up she complained of worsening symptoms and noted an external nasal deformity. She underwent complete revision ESS with a closed rhinoplasty performed only with external manual compression. Postoperative external examination demonstrated normal nasal contour (Figure 4).

### 4.3 Case 3

**FIGURE 5:**
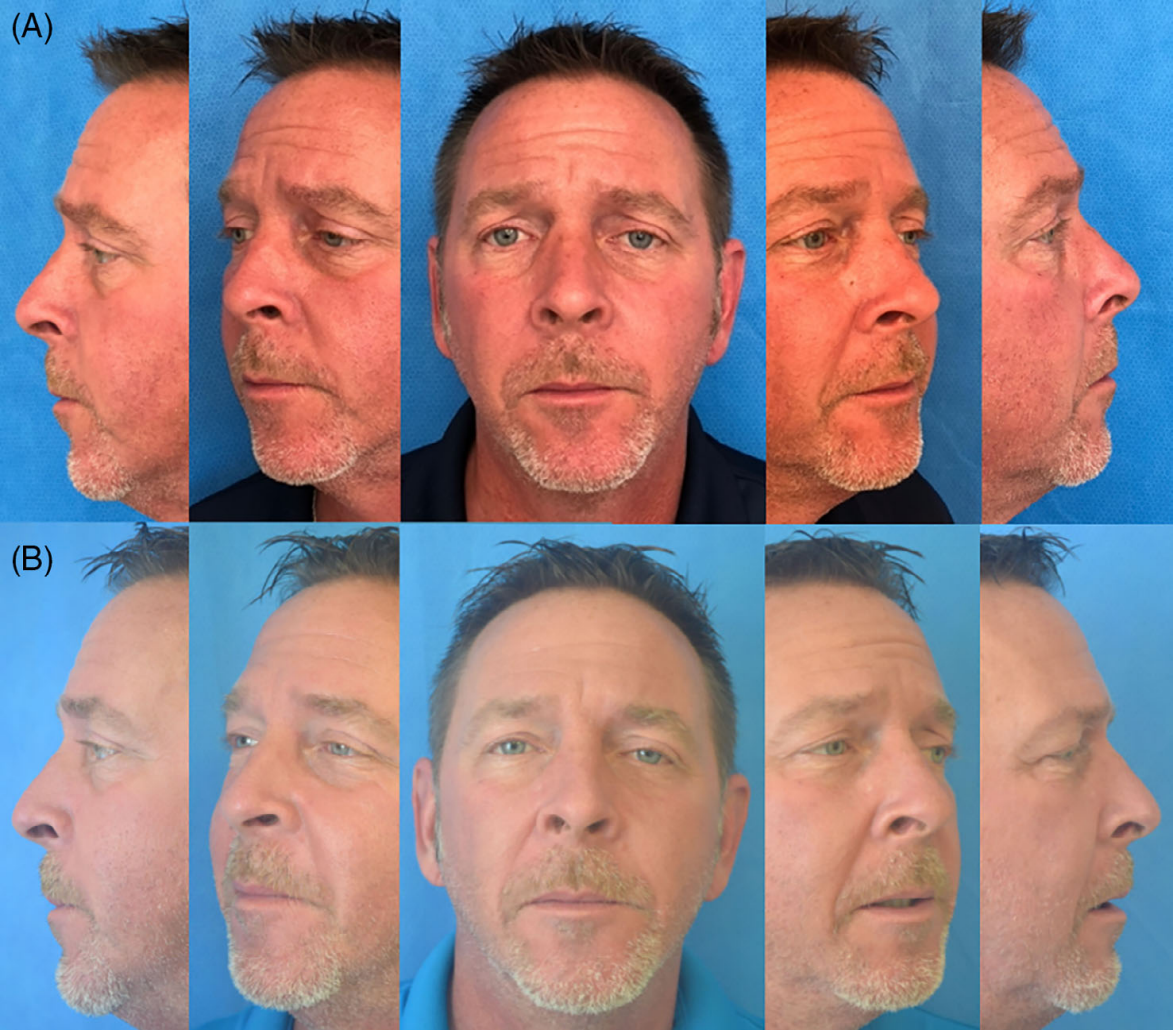
Case three: Preop (A) and one‐year postop (B)

A 51‐year‐old male without a history of asthma or ASA sensitivity presented with progressive nasal obstruction and right nasal asymmetry over 5 years. Exam revealed a tender nasal bone deformity in the right medial canthal region, septal deviation, and bilateral grade IV polyposis. On CT scan, bilateral frontal sinuses were completely opacified with rarefication of the right nasal bone and lateral displacement, while the other paranasal sinuses were largely spared. Because of the atypical presentation, a biopsy was performed that reported moderate eosinophilia and polyposis with no evidence of malignancy. Criteria for allergic fungal rhinosinusitis (AFRS) was not met. He underwent ESS and septoplasty, with closed rhinoplasty using manual reduction and a lateral osteotomy simply by pressing the osteotome into the nasal bone without a mallet. Postoperative follow‐up showed excellent nasal patency and normalization of external nasal cosmesis (Figure 5).

## 5 DISCUSSION

Classically, Woakes' disease was comprised of a constellation of severe nasal polyposis with broadening of the nasal bones, frontal sinus aplasia, bronchiectasis, and thickened mucus in children, frequently as a consequence of underlying disorders of mucociliary transport such as primary ciliary dyskinesia or cystic fibrosis.^3^ Since its characterization in the late 1800s, the condition has evolved in terms of diagnostic features, with the modern interpretation of “Woakes' disease” referring simply to CRSwNP leading to nasal bone rarefication and expansion, with young age and underlying genetic conditions unnecessary, and other local complications denoting progression in disease severity.^1^, ^4^

In this case series, two patients had a diagnosis of N‐ERD, however all three demonstrated eosinophilic disease. Recent evidence suggests that the degree of eosinophilia may be independently associated with severity of nasal polyposis,^5^, ^6^ at least in terms of imaging severity,^7^ though the presence of inflammatory cells may also play a key role in the bony resorption seen with this condition.

Indeed, while one often sees bony resorption in CRS, many patients conversely develop osteitis, a histopathological phenomenon often referred to synonymously as hyperostosis. The mechanism for this has not been conclusively identified, though is thought to be inflammatory and not infectious in nature.^8^, ^9^ Histopathologic evaluation has repeatedly demonstrated that ethmoid bone can be found in varying stages of resorption and osteoneogenesis in those with CRS,^10^, ^11^ suggesting that local disease characteristics play an important role in bone homeostasis.

There does not appear to be a preponderance for bony resorption in eosinophilic versus noneosinophilic disease. However, Laury et al. reported that the extracellular matrix protein periostin is significantly increased in AFRS tissue compared to CRS without nasal polyps (CRSsNP) and controls, and is correlated with bone erosion.^12^ The expression of periostin is upregulated by several cytokines and growth factors including IL‐4, IL‐13, TGF‐*β*, and TNF,^13^ all of which are secreted by eosinophils.^14^ Thus, periostin may both serve as marker of eosinophilic sinus disease and contribute to its varied bony pathology, and indeed Maxfield et al. have found periostin to be a potential biomarker of CRS endotype.^15^

The cytokine CCL11 (eotaxin 1) is a potent chemoattractant for eosinophils that is released by activated immune cells, including eosinophils themselves.^16^, ^17^ A recent study has reported an increase in CCL11 production by osteoblasts in response to inflammatory disease in vivo. A marked increase in osteoclast migration and bone resorption was consequently observed.^18^ From an otolaryngology perspective, nasal polyps are an abundant source of eotaxin, particularly in N‐ERD patients,^19^ and the proximity of these polyps to the adjacent nasal bones may hypothetically result in homeostatic dysregulation favoring bone resorption.

Pressure applied to the nasal bone because of increasing polyp burden may also be a significant factor in bone resorption. Mechanical loading can improve bone structure and strength.^20^ However, oral pathology literature suggests that there may be a pressure threshold, above which bone resorption occurs, particularly under continuous pressure loading.^21^, ^22^, ^23^

Despite the above theories, Woakes' disease remains an increasingly rare complication of a prevalent disease,^9^, ^24^ likely due to the earlier identification and management of genetic ciliary disorders and severe eosinophilic CRSwNP. Case reports have demonstrated that presentations vary widely in patient age and polyp etiology, and have not demonstrated consistency in management, particularly the correction of nasal deformities. Several studies made no mention of cosmetic repair.^25^, ^26^, ^27^ Decades ago, Pierre et al. reported employing autologous bone grafts to augment open rhinoplasty.^28^ In the maxillofacial surgery literature, Ueda et al. reported a midface degloving approach to perform their nasal osteotomies, though it should be noted that their case also necessitated correction of an anterior maxillary projection deformity.^29^ The Swiss otolaryngologist Abel‐Jan Tasman was the first to employ simultaneous closed rhinoplasty with digital compression at the time of endoscopic surgery,^30^ the same technique that we report in this series. Our experience with this simple technique has demonstrated excellent functional and cosmetic results, with subjective recovery of bone strength.

## 6 CONCLUSION

Woakes' disease remains relatively unknown, even amongst the otolaryngology community. The literature agrees that their refractory CRSwNP requires aggressive, targeted therapy to prevent further complications. However, the correction of their nasal pyramid expansion has been varied and inconsistent.

Simultaneous closed rhinoplasty with manual digital compression of the nasal bones is quick and easy to perform and leads to excellent symmetry of the nasal bones. Once the bone has been adequately rarefied to cause visible external expansion, osteotomies are often not necessary for adequate reduction but can easily be concurrently performed if needed.

## CONFLICT OF INTEREST

The authors declares there is no potential conflicts of interest.
